# The Impact of Digital Education on Communication Skills Among Medical Students in Ibn Sina University: A Cross-Sectional Online Survey

**DOI:** 10.7759/cureus.110260

**Published:** 2026-06-04

**Authors:** Ayman Subahi, Ali Al Ismaaiel, Eihab A Subahi

**Affiliations:** 1 Community Medicine, Ibn Sina University, Khartoum, SDN; 2 Internal Medicine, University of Toledo, Ohio, USA

**Keywords:** asynchronous learning, communication skills, digital education, distance education, medical education, medical students, online learning, synchronous learning

## Abstract

We distributed a web-based cross-sectional questionnaire to 446 medical students at Ibn Sina University. The questionnaire was based on a comprehensive review of the literature including, in particular, the questionnaire from First Aid for the USMLE Step 1 and a communication survey developed by the University of Louisville Ombuds Office, Louisville, KY. We concluded that online education has had a negative impact on social, personal, and family communication but a positive impact on academic communication. In our comparison of Sudanese and foreign students using patient-physician scenarios, both groups scored relatively low, but the foreign students performed better across most scenarios. This finding indicates that, when faced with the challenges posed by online education, the foreign students were able to demonstrate better communication skills in these scenarios than their Sudanese counterparts. We also found that the Sudanese medical students were less accustomed to using technology in their education and could be at a disadvantage when it comes to developing effective communication skills in an online learning environment.

## Introduction

Since the COVID-19 pandemic, digital education has become increasingly popular, with even third-world countries implementing online education. This educational transformation has affected the methods of acquiring knowledge in all disciplines. The impact of online education depends on numerous factors, including the nature of the subject being taught, the culture of the learners, the availability of a stable connection and smartphones, and many other intervening factors [1,2]. Communication skills are among the critical subjects for learners, especially those studying to be medical professionals, as a large portion of healthcare work depends on the ability to communicate effectively with patients [1-5].

In particular, communication skills enable healthcare workers to develop the appropriate strategies to deal with patients. Thus, some published guidelines include approaches to doctor-to-patient communication, a well-known example being the study by Cegala and Broz [4]. Similarly, Altohami et al. examined the disruption of clinical training and the psychological impact on specialty registrars as medical education has moved away from traditional formats [5]. The impact of delivering medical courses through online platforms has been debated in the literature, mainly because it depends on the culture of the students being researched [6,7]. In 2022, in response to the COVID-19 pandemic, the course “Clinical Topics in Global Health” at Harvard University was offered online for the first time, and a longitudinal research study in this context consisting of three online surveys concluded that e-learning can effectively replace traditional methods of education [1]. By contrast, a study of medical students in Gaza concluded that they did not have favorable experiences with online education and that action was necessary to overcome the challenges involved with this format [2].

Similarly, a study on the shift to online medical education during the COVID-19 pandemic on Egyptian medical students concluded that the impact of the shift was significant, with students reporting various limitations and challenges that needed to be addressed in order to realize the potential benefits of online platforms [3]. Currently, most universities in Khartoum, Sudan, depend, in part, on online-based education for most of the courses offered, and some universities, such as Alrazi University, are fully dependent on e-learning. We argue that previous studies are not necessarily generalizable to medical students at Ibn Sina University in Khartoum, Sudan, because of limitations that affect their validity. In particular, many of these studies tend to emphasize either the advantages or the disadvantages of online education. Thus, because these earlier studies focused on different populations and may suffer from selection bias and a lack of matching, and because, as discussed, the competence of doctors depends not only on their clinical skills but also on their communication skills and relationships with their patients, research on the impact of e-learning on the communication skills of medical students at Ibn Sina University is needed to maximize the benefits and minimize the harms associated with online coursework.

To address these issues, we first estimated the negative and positive impacts of digital education on communication skills from social, academic, family, and personal perspectives. The focus was on students in their fifth and sixth clinical years. We assessed students’ skills in communicating with patients using hypothetical hospital-oriented scenarios.

## Materials and methods

Design and settings

From January 10 to February 21, 2024, we distributed a web-based cross-sectional survey to medical students at Ibn Sina University using social media applications (Telegram, WhatsApp, and Facebook) and e-mail. Students across all academic years were offered the opportunity to participate. Because of our limited time and budget, we chose a cross-sectional study design to enable efficient collection of the data from a large population in a short period of time. In this way, we were able to explore the entire spectrum of experiences with online education and make comparisons across demographic and educational variables.

Sampling and participants

Ibn Sina University was established in 2000 in Khartoum on two campuses. As of June 2020, the total number of students registered in all of the faculties at the university was 6,060. Each year, approximately 2,000 students register at the university. Based on these data, we used Raosoft online software [8] to calculate the minimum recommended sample size, which was 362 participants. To confirm this figure, we double-checked sample size calculations using the traditional Cochran formula, with a 94.5% confidence level, a 0.05 margin of error, and a 50% response distribution:

X = Z² × p × (1 − p) / MOE² [9]

By the end of the data collection phase, on February 21, 2024, we had gathered a total of 446 responses. The inclusion criteria were registered medical students at Ibn Sina University who had completed one or more semesters of online classes. The exclusion criteria were respondents from non-medical faculties and those who had not completed at least one semester of online classes. The independent variable was digital education, and the dependent variable was communication skills.

Development of the survey instrument

The formulation of the questionnaire was based on a comprehensive review of the most recent literature regarding our research topic. We derived our questionnaire from two main authentic references. One was First Aid for the USMLE Step 1, 33rd edition, published at the beginning of 2023, in which the chapter on public health science includes a section dedicated to communication skills that provides patient-centered interviewing techniques and advice for communicating with patients with disabilities and managing challenging patient and ethical scenarios [10]. This is a commercially published textbook; the scenarios were adapted for educational research purposes. We used this questionnaire as the basis for formulating Section 4 of our questionnaire (full explanations of the scenarios with appropriate responses are included in the Appendix). The other main source that we used to formulate our questionnaire was the Communication Survey Questions developed and published by the University of Louisville Ombuds Office, Louisville, KY, an open-access resource publicly available on the University of Louisville Ombuds Office website [11].

The questionnaire consisted of four sections. Section 1 included an introduction page that provided information about the objectives and significance of our research and informed consent to participate. Section 2 consisted of an inquiry about sociodemographic characteristics, including the participants’ gender, faculty, academic year, and age. Section 3 included eight questions scored on a three-point Likert scale that assessed the impact of the transformation from traditional education to online education across four aspects (personal, family, academic, and social). Each participant was asked to choose either 1 = agree, 2 = neutral, or 3 = disagree. Section 4 included eight dichotomous questions that assessed the participants’ responses to challenging patient-physician scenarios, which were limited to either yes = appropriate response or no = wrong response. All of the questions were mandatory except for one open-ended question concerning the participants’ experiences with digital education tools, their perceived improvements in communication skills, and their opinions on the effectiveness of digital education in enhancing communication skills. This question was made optional to increase the response rate.

Validity and reliability of the instrument

The questionnaire and study protocol were tested on 45 students as part of a pilot study. They included 30 female students and 15 male students selected randomly across academic levels and faculties. Based on their feedback, we modified and adjusted the questionnaire to ensure that all of the participants could understand and answer the required questions. We chose 45 participants for this pilot study because a basic rule of thumb is to use 30 or more as an estimation parameter. Therefore, 45 participants were sufficient to demonstrate the instrument’s validity and reliability while also providing insightful information and an internal review of the questionnaire.

Data analysis

All of the collected data were extracted from Google Forms, transferred directly to an Excel spreadsheet (Microsoft Corp., Redmond, WA), and appropriately coded with numbers to make the data applicable to the statistical tests. All of the data were analyzed using IBM SPSS Statistics Version 26 (IBM Corp., Armonk, NY). The chi-square test was used to assess group differences in the patient-physician scenario responses. P-values < 0.05 were considered statistically significant. The analyses were restricted to the baseline participants (N = 446).

Ethical considerations

The study was conducted in accordance with the applicable ethical guidelines. Participation in the survey was completely voluntary. Informed consent was obtained from all of the participants beforehand, and they were informed that they had the right to withdraw at any point without any negative consequences. The confidentiality of the data was assured, and a coding system served to anonymize the participants’ data. The research purposes and objectives were explained to all of the participants in clear, simple language, and the results were reported ethically and transparently. The privacy and rights of the participants were respected.

## Results

Before the presentation of the results of this study, it is important to define two key terms. We used the term “foreigners” to refer to both international students and Sudanese students who had lived outside Sudan for more than one year. We used the term “Sudanese” to refer to Sudanese students who had spent no more than an entire year outside Sudan. These definitions were crucial for setting the parameters for the demographic groups in our study.

Characteristics of the sample

The sociodemographic data gathered for this study provided a comprehensive overview of the participants’ demographics. The study included a total of 446 participants, with a gender distribution of 33.9% men and 66.1% women. The Sudanese participants included 93 men (20.8% of the total participants) and 246 women (54.5% of the total participants), who together represented the majority of the participants. The foreign participants included 58 men (13.0% of the total participants) and 49 women (11.0% of the total participants). The age distribution of the participants was diverse, providing a broad perspective on the impact of online education across age groups. The 72 participants aged 17-19 years constituted 16.1% of the total, the 133 participants aged 20-22 years constituted 29.8%, the 202 participants aged 23-25 years constituted 45.3% of the total, and the 39 participants aged 26-30 years made up 8.7% of the total. The age and gender distribution of the participants are presented in Table 1.

**Table 1:** Age and gender distribution of the participants. Data are presented as N and %. Group differences between foreign and Sudanese students were assessed using the chi-square test (χ²). A p-value of <0.05 was considered statistically significant.

		Foreign	Sudanese	Total %	χ²	p-Value
Gender	Male	58	93	33.9%	24.85	<0.001
	Female	49	246	66.1%	24.85	<0.001
Age	17–19	6	66	16.1%	10.54	0.001
	20–22	43	90	29.8%	6.59	0.010
	23–25	91	111	45.3%	87.69	<0.001
	26–30	12	27	8.7%	0.71	0.400

The distribution of the participants by academic year is shown in Table 2. The 23 first-year students accounted for the smallest proportion of our study population (5.2% of the total). Overall, 30 second-year students accounted for 6.7% of the total, 49 third-year students accounted for 11.0%, and 94 fourth-year students accounted for 21.1%. Also, 211 fifth-year students accounted for 47.3% of the total, and 39 sixth-year students accounted for 8.7%. Notably, in Section 4 of our survey, which assessed patient-physician scenarios, we included only the fifth- and sixth-year medical students to ensure that these participants would have some communication skills and clinical exposure, which are typically gained in the advanced years of medical education.

**Table 2:** Academic year distribution of the participants. Data are presented as N and %. Group differences between foreign and Sudanese students were assessed using the chi-square test (χ²). A p-value of <0.05 was considered statistically significant.

Academic Year	Foreign	Sudanese	Total %	χ²	p-Value
First year	0	23	5.2	6.33	0.012
Second year	1	29	6.7	6.36	0.012
Third year	13	36	11.0	0.07	0.792
Fourth year	27	67	21.1	1.15	0.283
Fifth year	55	156	47.3	0.74	0.389
Sixth year	11	28	8.7	0.20	0.654

We also included participants across various faculties (Table 3). Most of the participants, 359 (80.5%), were from the Faculty of Medicine. Of the remainder, 39 (8.7%) were from the Faculty of Dentistry, 27 (6.1%) were from the Faculty of Pharmacy, 16 (3.6%) were from the Laboratory Sciences, and 5 (1.1%) were from the Faculty of Nursing.

**Table 3:** Faculty distribution of the participants. Data are presented as N and %. Group differences between foreign and Sudanese students were assessed using the chi-square test (χ²). A p-value of < 0.05 was considered statistically significant.

Specialty	Foreign	Sudanese	Total %	χ²	p-Value
Medicine	80	279	80.5	2.48	0.115
Dentistry	12	27	8.7	0.71	0.400
Pharmacy	8	19	6.1	0.23	0.635
Laboratory sciences	4	12	3.6	0.00	1.000
Nursing	3	2	1.1	1.88	0.171

Impact on family, personal, academic, and social aspects

**Figure 1:**
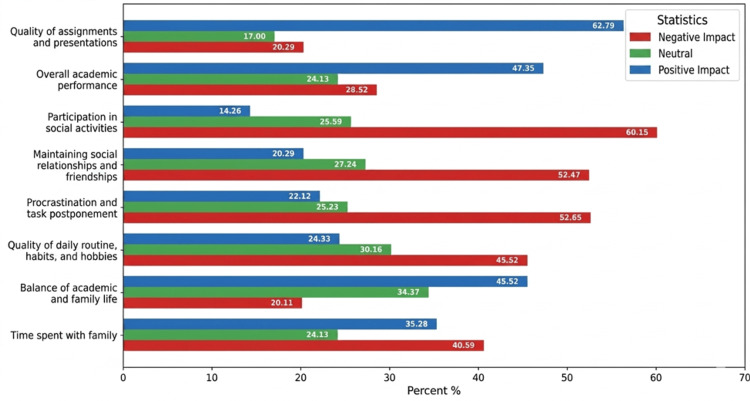
Impact of online education on the family, personal, academic, and social aspects of communication among respondents (N = 446). Source: the authors’ survey data, 2024.

We assessed the impact of online education on the four main aspects of medical students’ lives (academic, social, personal, and family) by formulating the eight questions presented in Figure 1. We combined the responses of both the foreign participants and the Sudanese participants because they were closely aligned.

The first two questions assessed the social aspect by inquiring about social interaction skills with peers and the ability to communicate in social settings. Most of the participants, 268 (60.1%), reported a negative impact on participation in social activities, though most, 234 (52.5%), also reported maintaining social relationships and friendships. These findings highlight the need for online education platforms to incorporate features that facilitate social interaction.

Regarding family communication, we found that online education had a predominantly negative impact. Specifically, 90 (20.1%) of the participants reported a negative impact on balancing academic life with family life, and 181 (40.6%) reported a negative impact on the amount of time spent with family. These results indicate that online education may create barriers to family communication, which is important for maintaining a healthy work-life balance.

Regarding the personal aspect, the responses revealed a similar trend. Significant proportions of the participants reported a negative impact on procrastination and postponing tasks (n = 235, 52.7%) and the quality of their daily routine, habits, and hobbies (n = 203, 45.5%).

The last two questions assessed the academic aspect. Most of the participants reported a positive impact of online education on their academic communication skills. Specifically, 280 (62.7%) reported a positive impact on the quality of their assignment presentations, and 211 (47.3%) reported a positive impact on their overall academic performance. These results suggest that online education may enhance certain academic skills, especially academic communication.

Patient-physician communication skills

A total of 66 foreign medical students in their fifth and sixth years were selected for this part of the study. To ensure a balanced comparison, equivalent numbers of Sudanese medical students were randomly selected from the same academic years. Section 4 of our questionnaire, which was specifically designed to evaluate the participants’ skills in patient-physician scenarios, served to assess these selected groups. Each question in this section presented a hypothetical patient-physician scenario, and the participants were asked to choose between an appropriate response and a wrong response (Table 4; the appropriate responses are included in the Appendix). The results, presented in Table 4, revealed significant differences in the responses of the Sudanese and the foreign medical students.

**Table 4:** Percentage of appropriate responses in clinical scenarios for Sudanese and foreign participants. Data are presented as percentages (%). Group differences were assessed using the chi-square test (χ²) with Yates’ continuity correction. A p-value of <0.05 was considered statistically significant.

Clinical Scenario	Sudanese (%)	Foreign (%)	χ²	p-Value
1. Family members asking for patient’s prognosis	59.1	80.3	6.06	0.014
2. Suicidal patient	9.1	22.7	3.62	0.057
3. Seven-year-old boy feeling responsible for losing a sister to cancer	27.3	42.4	2.70	0.100
4. Patient desiring an unnecessary procedure	19.7	51.5	13.22	<0.001
5. Dependent patient with injuries inconsistent with the caretaker’s story	27.3	81.8	37.43	<0.001
6. Patient not following the medical plan	31.8	27.3	0.15	0.703
7. Patient angry about a long waiting time	42.4	45.5	0.03	0.861
8. Invasive test performed on the wrong patient	4.5	37.9	19.99	<0.001

The first scenario involved family members asking for information about a patient’s prognosis. In this scenario, 39 (59.1%) of the Sudanese medical students chose the appropriate response compared with 80.3% of the foreign students. The second scenario involved a suicidal patient; only 6 (9.1%) of the Sudanese students chose the appropriate response compared with 15 (22.7%) of the foreign students. The third scenario involved a seven-year-old boy who feels responsible for losing a sister to cancer; 18 (27.3%) of the Sudanese students chose the appropriate response compared with 28 (42.4%) of the foreign students. The fourth scenario involved a patient desiring an unnecessary procedure; 13 (19.7%) of the Sudanese students chose the appropriate response compared with 34 (51.5%) of the foreign students. The fifth scenario involved a dependent patient presenting with injuries inconsistent with the caretaker’s story; 18 (27.3%) of the Sudanese students chose the appropriate response compared with 54 (81.8%) of the foreign students. The sixth scenario involved a patient not following the medical plan; 21 (31.8%) of the Sudanese students chose the appropriate response compared with 18 (27.3%) of the foreign students. The seventh scenario involved a patient angry about a long waiting time; 28 (42.4%) of the Sudanese students and 30 (45.5%) of the foreign students chose the appropriate response. The final scenario involved an invasive test being performed on the wrong patient; only 3 (4.5%) of the Sudanese students chose the appropriate response compared with 25 (37.9%) of the foreign students.

Overall, while both groups scored relatively low, the foreign students performed better across most of the scenarios. This result indicates that, when faced with the challenges posed by online education, the foreign students demonstrated better communication skills in these patient-physician scenarios than their Sudanese counterparts. These findings emphasize the importance of effective communication in patient care and the need for targeted interventions to enhance communication skills among medical students in online coursework.

## Discussion

The aim of this study was to assess the impact of online education on communication skills among students at Ibn Sina University. The findings suggest that online education has a predominantly negative impact on the social, personal, and family aspects of communication skills but a positive impact on the academic aspect. Similarly, in a study of students at the American University of Madaba in Jordan, online learning had an overall negative impact on communication between instructors and students because of the isolated environment and the resulting lack of competitiveness and motivation [7]. Similarly, in a systematic review of e-learning in health professions education during the COVID-19 pandemic by Naciri et al., students generally perceived e-learning positively in terms of flexibility and accessibility, but they had significant concerns regarding the loss of hands-on training and the development of interpersonal communication [12].

The negative impact of online learning on social communication observed in our study, in which 268 (60.1%) of the participants reported reduced engagement in social activities and 234 (52.5%) reported difficulty maintaining relationships, is consistent with broader findings in the literature. A scoping review of the use of digital technology to develop communication skills in undergraduate and postgraduate medical education published in JMIR Medical Education in 2026 found that while virtual patient simulators and live-streaming technologies have expanded rapidly, they cannot fully replicate the nuanced social interactions that occur in face-to-face educational settings [13]. This finding underscores the concern that digital education, while offering flexibility, may inadvertently erode the social competencies that are essential for healthcare professionals.

Our finding that online education positively impacted academic communication (with 280 of the participants, 62.7%, reporting improvement in assignment presentations) deserves attention. This finding may be attributable to the structured nature of online platforms, which often require students to prepare and present materials in organized digital formats. Dewi et al. found that structured communication skills curricula, even when delivered through blended learning approaches, can yield positive outcomes in specific competency areas provided that feedback mechanisms are incorporated [14]. This finding suggests that the academic domain may benefit from the structured requirements of online learning, even as other communication domains suffer.

Regarding the patient-physician communication scenarios, our finding that both the Sudanese students and the foreign students scored relatively low across most of the scenarios raises concerns about the overall preparedness of medical graduates for clinical communication. The strikingly low appropriate response rate among the Sudanese students for the suicidal patient scenario (n = 6, 9.1%) and the wrong-patient invasive test scenario (n = 3, 4.5%) indicates critical gaps in communication training related to patient safety and crisis management. Zerbini et al. evaluated a new communication curriculum at the University of Augsburg and found that longitudinal, integrated communication training significantly improved students’ self-assessed skills and confidence and suggested that structured curricula may address such gaps [15]. Our results further support the need for dedicated communication skills training that extends beyond traditional lecture-based delivery.

 Therefore, the consistent performance gap between the foreign students and the Sudanese students across most of the scenarios (with the notable exception of Scenarios 6 and 7) may reflect differential exposure to technology-enhanced learning prior to enrollment at Ibn Sina University. The students categorized as foreign, having lived outside Sudan for more than one year, may have had greater prior exposure to digital educational tools and online communication platforms than the students categorized as Sudanese, and this exposure may have provided the foreign students with a relative advantage in navigating online learning environments. This interpretation is consistent with the findings of Alawamleh et al., who reported that students less accustomed to online platforms experienced greater communication difficulties than students who were more accustomed to online platforms [6]. Unfortunately, the assessment of the academic aspect was not entirely accurate because Ibn Sina University does not use a grade point average (GPA) grading system. This limitation to the study design highlights the need for globally valid measures of academic performance to facilitate future research at Ibn Sina University (e.g., a four-point scale or percentage-based grading system).

This finding should serve as a strong motivation to initiate pre-admission courses that train newly accepted students in the use of online lectures and platforms such as Moodle, which was the main platform used at Ibn Sina University during the first year of the COVID-19 pandemic. This conclusion is consistent with the findings in a study by Lucey and Johnston, which reported that the sudden transition to online education caused by the coronavirus pandemic has impacted several aspects of medical education, including the patient-doctor relationship, which is highly dependent on communication skills [7].

Limitations

This study has several limitations that should be considered when interpreting the findings. First, the cross-sectional design limits the ability to establish causal relationships between online education and communication skills; a longitudinal design would provide stronger evidence of temporal associations. Second, the use of a convenience sampling method involving social media platforms may have introduced self-selection bias, in that students who were more comfortable with technology may have been more likely to participate. Third, the questionnaire relied on self-reported perceptions and dichotomous responses to hypothetical scenarios that may not accurately reflect real-world clinical communication competencies. Fourth, the lack of a standardized GPA grading system at Ibn Sina University limited our ability to assess the academic dimension. Fifth, the definition of “foreigner” in this study, which included Sudanese students who had lived abroad for more than one year, may conflate exposure to international education with other cultural or socioeconomic factors. Lastly, this study was limited to a single university in Khartoum, and thus the findings may not be generalizable to other Sudanese or regional institutions. Future studies should consider the use of multi-institutional designs, validated communication assessment tools (such as objective structured clinical examinations), and mixed-methods approaches to provide a more comprehensive evaluation of the impact of digital education on communication skills.

## Conclusions

The findings presented here indicate that online education may have a differential impact on communication skills because of variation in students’ familiarity and comfort with technology. This differential impact may be detrimental to the healthcare system in the future, as the first generation of students who used online education throughout their academic years will graduate in the coming years, at which point the consequences and impact of this transition will be evident. Specifically, it appears that Sudanese medical students, who may be unaccustomed to using technology in their education, could be at a disadvantage when it comes to developing effective communication skills in online learning environments. Future research can determine which online education system best fits Sudanese medical students and the best type of training for both tutors and students to mitigate the challenges associated with this education mode and enhance its positive aspects. Such research can help ensure that the doctors of tomorrow are not only academically proficient but also effective communicators.

## Appendices

Appendix

Study Questionnaire

Section 1: Introduction and Informed Consent

Dear participant, you are invited to participate in a research study titled “The Impact of Digital Education on Communication Skills Among Medical Students at Ibn Sina University.” This study aims to assess how the shift from traditional to online education has affected the communication skills of medical students. Your participation is entirely voluntary, and your responses will remain anonymous and confidential. By proceeding with this questionnaire, you provide your informed consent to participate in this study. You may withdraw at any time without any consequences.

Section 2: Sociodemographic Characteristics

1. Gender: Male / Female

2. Age: 17-19 / 20-22 / 23-25 / 26-30

3. Nationality: Sudanese / Foreign

4. Faculty: Medicine / Dentistry / Pharmacy / Laboratory Sciences / Nursing

5. Academic Year: First / Second / Third / Fourth / Fifth / Sixth

Section 3: Impact of Online Education on Communication Skills (Likert Scale: 1 = Agree, 2 = Neutral, 3 = Disagree)

1. Online education has negatively affected my participation in social activities.

2. Online education has negatively affected my ability to maintain relationships and communicate in social settings.

3. Online education has negatively affected my ability to balance academic responsibilities with family communication.

4. Online education has negatively affected the time I spend with my family.

5. Online education has led to increased procrastination and postponing of tasks.

6. Online education has negatively affected my daily routine.

7. Online education has positively affected my ability to deliver presentations and assignments.

8. Online education has positively affected my academic performance.

Section 4: Patient-Physician Communication Scenarios (Appropriate Response / Wrong Response)

The following are the appropriate responses for each patient-physician scenario used in Section 4 of the questionnaire, derived from First Aid for the USMLE Step 1, 33rd edition (see Table 4 for the student response rates).

1. Family members ask for information about the patient’s prognosis: avoid discussing issues with relatives without the patient’s permission.

2. Patient is suicidal: assess the seriousness of the threat. If the patient is actively suicidal with a plan, suggest remaining in the hospital voluntarily; the patient may be hospitalized involuntarily if needed.

3. A seven-year-old boy feels responsible for losing a sister to cancer: at five to seven years of age, children begin to understand that death is permanent, all life functions end completely at death, and everything that is alive eventually dies. Provide a direct, concrete description of his sister’s death. Avoid clichés and euphemisms. Reassure the boy that he is not responsible. Identify and normalize his fears and feelings. Encourage play and healthy coping behaviors (e.g., remembering her in his own way).

4. Patient desires an unnecessary procedure: attempt to understand why the patient wants the procedure and address the underlying concerns. Do not refuse to see the patient or refer the patient to another physician. Avoid performing unnecessary procedures.

5. A dependent patient presents with injuries inconsistent with the caretaker’s story: document detailed history and physical examination. If possible and appropriate, interview the patient alone. Provide any necessary medical care. If suspicion remains, contact the appropriate agencies or authorities (e.g., child or adult protective services) for an evaluation. Inform the caretaker of your obligation to report. Physicians are required by law to report any reasonable suspicion of abuse, neglect, or endangerment.

6. Patient does not follow the medical plan: determine whether there are financial, logistical, or other obstacles preventing the patient’s adherence. Do not coerce the patient into adhering or refer the patient to another physician. Schedule regular follow-up visits to track patient progress.

7. Patient is angry about the long time spent in the waiting room: acknowledge the patient’s anger, but do not take the patient’s anger personally. Thank the patient for being patient and apologize for any inconvenience. Stay away from efforts to explain the delay.

8. An invasive test is performed on the wrong patient: regardless of the outcome, a physician is ethically obligated to inform a patient that a mistake has been made.
